# Multiphoton Microscopy to Visualize Live Renal Nerves in Reanimated Kidney Blocks

**DOI:** 10.3390/jimaging11020056

**Published:** 2025-02-13

**Authors:** Joerg Reifart, Patrick T. Willey, Paul A. Iaizzo

**Affiliations:** 1 Visible Heart® Laboratories, Department of Bioengineering, University of Minnesota, Minneapolis, MN 55455, USA; 2 Cardiovascular Department, School of Medicine, University of Minnesota, Minneapolis, MN 55455, USA; 3 University Imaging Center, University of Minnesota, Minneapolis, MN 55455, USA

**Keywords:** renal denervation, isolated perfused kidney, multiphoton microscopy, renal artery

## Abstract

Renal denervation to treat arterial hypertension is growing in adoption but still shows inconsistent results. Device improvement is difficult, as there is currently no way to study the immediate success of renal denervation devices in living tissue. In an effort to visualize live renal nerves surrounding their arteries using multiphoton microscopy, kidney pairs were explanted from Yorkshire pigs. They were maintained viable with a pulsatile perfusion apparatus using Visible Kidney™ methodologies, in which blood is replaced by a modified, oxygenated, and warmed (37 °C) Krebs–Henseleit buffer. The block resection allows catheter placement for nerve ablation treatment. Subsequently, the kidney block was disconnected from the perfusion system and underwent multiphoton microscopy (Nikon A1R 1024 MP). A total of three renal blocks were imaged using this model. Using 780 nm excitation for autofluorescence, we were able to selectively image peri-arterial nerves (2.5–23 μm diameter) alongside arteriolar elastin fibers (1.96 ± 0.87 μm; range: 0.3–4.27) at 25× magnification at a pixel size of 1.02 µm). Autofluoresecence was not strong enough to identify nerves at 4× magnification. There was a high but variable signal-to-noise ratio of 52.3 (median, IQR 159). This model may be useful for improving future physician training and innovations in renal denervation technologies.

## 1. Introduction

Renal denervation is experiencing renewed interest as a treatment for arterial hypertension that is refractory to medication, following the positive results from recent sham-controlled trials [1,2]. An effective ablation of the peri-arterial renal nerves is crucial for reliable results. However, anatomical variabilities and limitations in our understanding of the factors affecting renal denervation remains challenging [3,4].

Recently, multiphoton microscopy has emerged as a method for label-free color imaging of nerves [5,6,7] and a method to sustain the viability of an explanted kidney block has been developed [8]. In this study, we sought to combine these techniques to create a model and imaging platform capable of potentially assessing the effects of renal denervation interventions in reanimated porcine and human kidney blocks.

## 2. Materials and Methods

The specimens used in this study were obtained from Yorkshire pigs after acute preclinical studies. The study protocol was approved by the Institutional Animal Care Committee of the University of Minnesota and was compliant with the National Research Council’s 2011 Guidelines for the Care and Use of Laboratory Animals.

**Figure 1:**
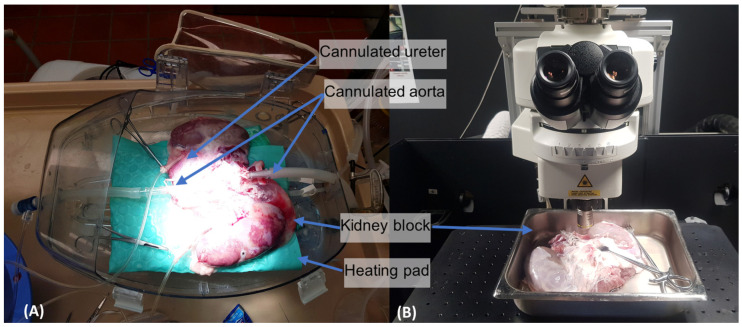
(**A**) Shows the Visible Kidney apparatus: the renal block is maintained in a modified OCS™ Lung housing capsule (TransMedics Inc., Andover, MA, USA). The kidney block rests on a heating mat and is connected to a pulsatile flow system continuously running oxygenated perfusate. The cannulated ureter allows for continuous urine output measurements. The tube cannulating the proximal aorta (right) carries the oxygenated perfusate. The distal tube cannulating the distal aorta (left) provides access for renal denervation catheters. (**B**) Next, the given kidney block was positioned under multiphoton microscope, partially submerged.

Kidney block preparation:

After the termination of the given preclinical study, both kidneys were explanted en-bloc with their respective arterial and venous supplies connected to the inferior vena cava and abdominal aorta; following the Visible Kidney protocol [8]. Each kidney block was rinsed and kept viable using Visible Kidney technologies. In short, the distal abdominal aorta was ligated, while the proximal abdominal aorta was cannulated and connected to the perfusion system. Venously returning perfusate was collected in the perfusate tank. The organs were then kept viable by providing perfusion with an oxygenated modified Krebs solution from the tank, kept at 37 degrees Celsius, using a pulsatile pump (Harvard Apparatus Model 1423 PBP, Holliston, MA, USA)) set at 60 beats per minute. The kidney blocks were maintained on the Visible Kidney apparatus until the multiphoton microscopy suite was available (Figure 1A). Upon availability, the specimens were disconnected from the apparatus and brought to the microscopy suite submerged in 4 L of modified Krebs solution.

Multiphoton microscopy:

Each sample was imaged on an FN1 upright Nikon microscope equipped with an A1R HD confocal scan head, non-descan GaAsP detectors in the epi direction, and a Nikon Plan Apo LWD 25×/1.1NA water dipping objective (Figure 1B). The tissue of interest was excited with a Spectra-Physics 15W Mai Tai eHP tunable IR laser tuned to 780 nm. Images were scanned unidirectionally in Galvano mode, and the frame sizes were 512 × 512. Nerve fiber autofluorescence signals were gathered from 470 to 550 nm. Z-stacks were acquired using a Prior Z-drive with a step size of 2 µm.

Image analysis:

ImageJ 1.54f (Wayne Rasband and contributors, National Institutes of Health, Madison, WI, USA) was used for analyses and measurements. Images were color coded and merged for the final images. For signal-to-noise ratios (SNRs), the autofluorescence signals from nerves were calculated using manual selections of circular 10–50 µm^2^ ROIs (depending on nerve size), compared to ROIs of equal sizes in the surrounding tissue. Elastin fiber and nerve diameters were measured throughout multiple slices of the same volume/stack. The results are presented as mean ± standard deviations or median and IQR as appropriate.

## 3. Results

Three kidney blocks were successfully explanted, maintained on the organ care system, and scanned. Multiphoton microscopy revealed brightly autofluorescent fibrous structures in a range of 0.8–23.5 μm diameter range.

**Figure 2:**
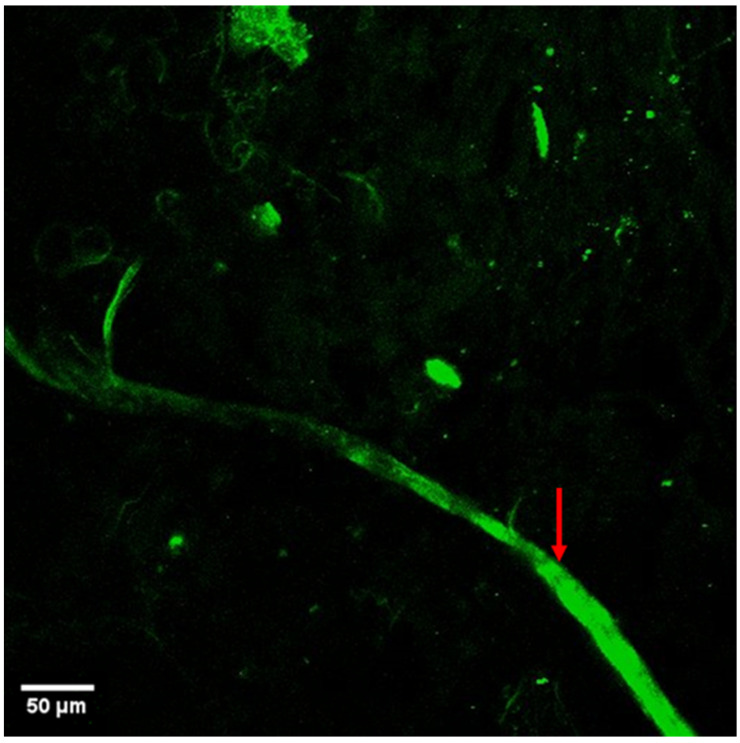
The autofluorescence imaging of a ranching renal nerve in the arterial space (marked by red arrow). Main nerve diameter around 25 µm with small branches as small as 2.5 µm.

Autofluorescent fibrous structures > 4 μm exhibited branching patterns characteristic of nerve morphology and were identified as nerves (Figure 2).

**Figure 3:**
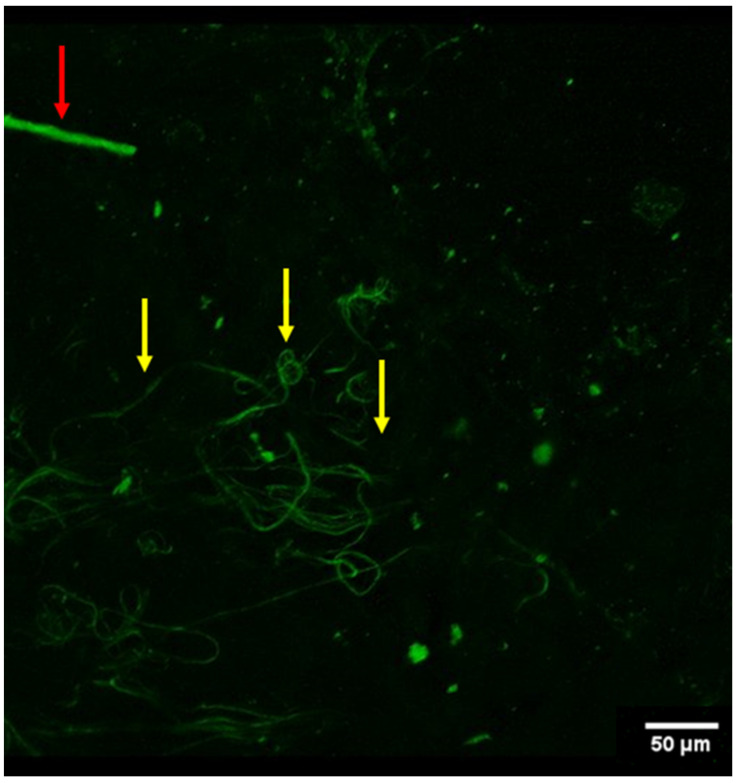
Conglomerate of what appear to be elastin fibers (yellow arrows) in the arterial vessel wall (viewed from autofluorescence). Also noted was a singular nerve fiber (red arrow) in the periphery of the field of view.

In contrast, non-branching, looping structures < 4 μm were identified as elastin fibers (Figure 3, Video S1). Elastin fibers typically appear as thin, wavy, or curly structures that form a dense, interconnected network within the arterial wall, contributing to vascular elasticity. The scanned volumes encompassed 520 μm × 520 μm × 106 μm (width × height × depth, Voxel size of 2.49 × 2.49 × 6.3 μm^3^). The median scanning time was 144.19 s (range: 52.3–284.56).

The average signal-to-noise ratio for nerves was 52.3 (median, IQR 159); indicating substantial variability.

At lower magnifications, autofluorescence elicited by the 780 nm laser was not strong enough to distinguish nerves. The number of nerve strands per mm^2^ field of view varied, and elastin fibers in the renal artery’s media exhibited significant autofluorescence (Table 1).

The multiplane scrolling feature of the microscope, enabled more detailed, 3D analyses of the renal nerve structures.

Multiphoton microscopy was able to identify, on average, 1.14 ± 0.8 renal nerve strands per field of view. Alongside renal nerves, elastin fibers of the renal artery media also elicited autofluorescence (Figure 2).

## 4. Discussion

Our findings suggest a potential utility of multiphoton microscopy for real-time imaging of renal nerves in perfused large mammalian kidney blocks. Despite the limited imaging depth (approximately 500 μm), this technique holds potential for evaluating renal denervation technologies, especially considering the peri-arterial locations of nerve bundles. Importantly, the ability to image in three dimensions provides a more comprehensive view of the nerves targeted during denervation procedures.

Currently, in order to verify effective nerve ablation, norepinephrine levels and histomorphometric techniques (staining and analyses of tissue slices) have been employed [9,10,11]. Yet, these techniques do not provide immediate feedback: thus, how selective and complete the given ablative treatment remains unclear. There are ongoing efforts to track ablation outcomes in live nerves by recording evoked action potentials; to date translation of such methods to renal nerve physiology has been difficult [12].

To our knowledge, the technique presented here is the first to show a potential path to refine renal denervation technologies. Notably, previous histological data in humans did not assess nerve diameters below 50 micrometer (which are exclusively presented here), the majority of renal nerves are found within 4 mm of the artery surfaces, with a tendency to have smaller diameter nerves in the closest proximities to these arteries [13]. While the employed methodologies signal-to-noise ratio was good overall, the substantial variability that was seen is not fully explained by nerve or elastin fibers traveling orthogonally to the z-plane: i.e., other tissues may also exhibit autofluorescence.

Future experiments are planned to allow us to test percutaneous renal denervation systems in viable human kidney blocks.

It should be noted, that there may be significant limitations to our described methods, and more work is required to confirm our following findings. First, the short viability window of the renal nerves requires uninterrupted workflow and immediate access to microscopy equipment, and the limited field of view requires multiple images to cover larger areas. Additionally, the structures were identified based on morphologies and previously described multiphoton microscopy of nerves, which had been validated with histopathological confirmation (6). The small fibers in the 1–5 µm range were assumed to be elastin fibers. Note, nerve fibers of the same diameter would only be morphologically distinguishable. For real-time analysis machine learning models (e.g., YOLO (You Only Look Once), SAM2 (Segment Anything Model) or custom-trained CNNs (convolutional neural networks)) could be employed to accurately identify nerves before ablation. Despite these challenges, multiphoton microscopy offers a promising platform for assessing outcomes and improving the designs of renal denervation technologies.

Note, due to the small sample size to date, future research is needed to expand on this area of investigation; i.e., whether or not, the real-time recording of renal ablation is indeed feasible. As multiphoton imaging has been used to selectively visualize spermatic nerve in vivo in a mouse model [6], developing this method for use in larger animals seems highly feasible.

## 5. Conclusions

Multiphoton microscopy, combined with the use of Visible Kidney™ methodologies, provides a novel research approach for visualizing renal nerves post-therapy in real time. These methodologies could facilitate the verifications of renal nerve ablation, aiding in physician training and improving future innovations in renal denervation technologies.

## Figures and Tables

**Table 1:** Nerve count and diameters.

Kidney Specimen No.	No. of Nerves per Field of View	Maximal Diameter (μm)	Minimal Diameter (μm)	Elastin Diameters (μm) ¹
1	2	13.9	5.2	3.16 ± 0.7
2	1	7.7	23.1	1.70 ± 0.69
3	1	17.1	11.8	NA

^1^ Mean ± standard deviation.

## Data Availability

The raw data supporting the conclusions of this article will be made available by the authors upon request.

## Supplementary Materials

The following supporting information can be downloaded at: https://www.mdpi.com/article/10.3390/jimaging11020056/s1, Video S1: Scan through renal artery tissue revealing a web of fibers morphologically consistent with elastin.
